# Intrarectal Foreign Body Detected at CT Scanner Investigating an Abdominal Syndrome

**DOI:** 10.1155/2019/9134735

**Published:** 2019-02-10

**Authors:** Florina Popa, Madalina Grigoroiu, Monica Elia Georgescu

**Affiliations:** ^1^Department of General Surgery, University of Medicine and Pharmacy “Iuliu Hatieganu”, Cluj-Napoca, Romania; ^2^Department of Morphological and Functional Sciences, “Dunarea de Jos” University of Galati, Faculty of Medicine and Pharmacy, Galati, Romania

## Abstract

The field of general surgery offers many different pathologies, cases, and situations for which the general surgeons should be competent in diagnosis as well as treatment and management, including operative intervention. Most situations are complicated by delayed admission to the hospital due to the embarrassment of patient and inability to obtain satisfactory anamnesis. This article reviews the use of computed tomography as a problem-solving tool in the identification, localization, and presurgical planning for extracting the rectal foreign object.

## 1. Introduction

The introduction of foreign bodies (FB) through the anus is a well-described phenomenon that still remains a curiosity and a taboo nowadays. Their particularity consists of being very diverse and unusual. It is a matter of time before every surgeon will have to deal with such a case. Foreign bodies may lead to perforation, obstruction, or infection. The extraction of foreign objects requires ingenuity. Since in most situations the surgeon is unaware of its consistence, imaging is the key to solve the problem. Computed tomography helps us to locate the foreign body and determine its relationship to surrounding structures and its depth of involvement.

## 2. Case Presentation

A 33-year-old woman presented to the emergency services for pain in the lower abdomen and anorectal pain. A detailed medical history of the patient revealed that her partner had inserted a foreign object into her rectum to achieve sexual satisfaction. The patient stated that she had not seen the foreign object and she did not know the nature of the material.

**Figure 1:**
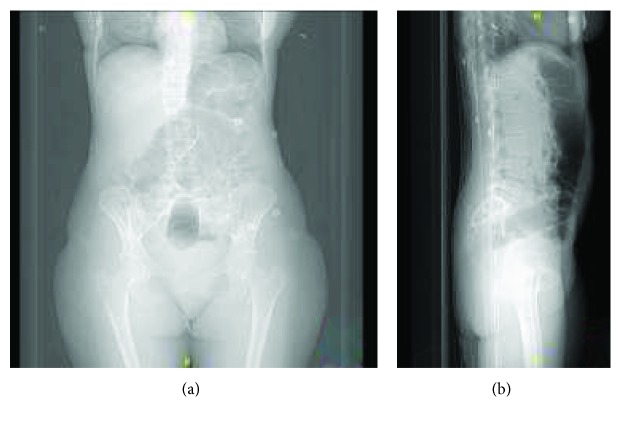
Standard frontal (a) and lateral (b) radiographs reveal the foreign body.

On physical examination, the abdomen was relaxed but at the palpation a hard object could be felt. Complete blood cell count (CBC) and biochemical parameters were within a normal range. On digital rectal examination, the base of the object was palpated as a solid object 8-9 cm proximal to the anus. Standing abdominal radiographs of the patient were obtained in an emergency department for differential diagnosis and showed a bottle in the rectum without any evidence of free air or air-fluid levels (Figure 1).

**Figure 2:**
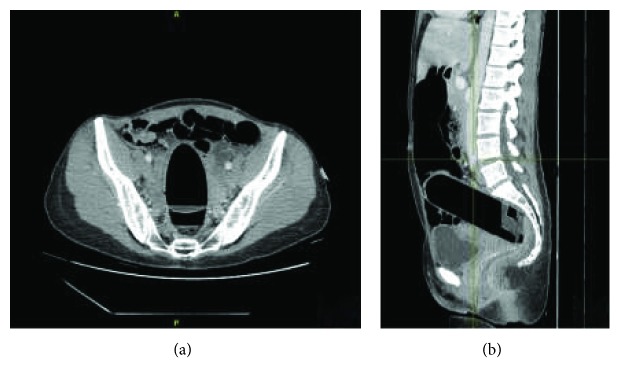
The visualization of the foreign body by CT: axial view (a) and sagittal view (b).

To obtain accurate information regarding the nature and location of the foreign object, the relationships with surrounding tissues, and potential complications, a CT was done (Figure 2).

**Figure 3:**
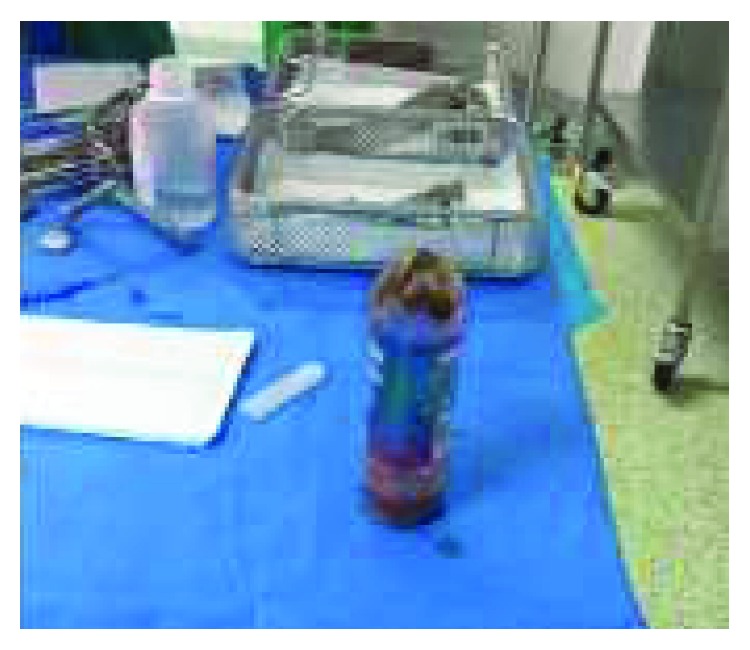
Surgically extracted rectal foreign object.

A 17 cm foreign body is viewed at the rectosigmoid level. The thickened appearance and hyperemia of the rectal walls indicates an associated proctitis. The patient was transferred to an operating room, the anal canal was dilated under general anesthesia, and the object was removed manually by pressing on the abdomen. Digital transanal extraction was successful after 45 minutes, when a total relaxation has been achieved and by applying bimanual continuous pressure on the anterior abdominal wall. The extracted object consisted of a lubricant gel tube (Figure 3). The postoperative period was uneventful.

## 3. Discussion

Rectal foreign bodies, in most cases, impose challenging strategies in order to remove it safely and an adequate management of care conditions during the convalescent period. In most cases, patients present several hours to days after the placement of the rectal foreign body, and on occasion, the foreign body has even been successfully removed, but the patient has delayed symptoms of bleeding, perforation, or even incontinence [1, 2]. Therefore, imaging is needed to diagnose the patient as quickly as possible. Imaging is effective at detecting most foreign bodies as well as aiding in their removal by clearly localizing the object of interest within the tissue. Various imaging modalities, such as conventional plain radiographs, CT, MRI, and ultrasonography, are used to detect foreign bodies. Conventional plain radiography is usually the preferred imaging method for detecting foreign bodies [3]. CT works similarly to radiography, but it has an improved ability to differentiate tissue densities, allowing for better visualization of foreign body-related complications because of its ability to provide volumetric information and detailed spatial resolution of anatomy and pathology. It comes with the added benefit of being able to provide a more accurate three-dimensional localization of the foreign body.

To exclude a possible perforation or peritonitis which leads to acute abdomen, abdominal radiographs were done followed by a CT scan. The role of CT in these cases is not limited to helping detect the foreign body and its exact location, but it includes helping identify possible complications and orient you in choosing the appropriate surgical technique to remove the foreign body.

Different operative techniques are used to remove rectal foreign bodies. Acute abdomen due to rectal or colonic perforation should be excluded [4]. The chosen procedure depends on the type of object, location of the RFB, time from insertion to presenting to the emergency room, symptoms of the patients, and the surgeon's skills. Laparotomy or laparoscopy is the last step when all conservative methods failed or the patient presented with symptoms of an acute abdomen or in the case of perforation [5].

Therapeutic options for patients without signs of perforation are manual extraction, endoscopic extraction, TAMIS extraction, laparoscopic or open advancement with transanal extraction, and laparoscopic or open transmural extraction [6].

In our case, deeper sedation assured the relaxation needed to remove the foreign body. Downward pressure on the object in the left iliac fossa greatly aids in moving the object toward the rectum and stabilizing it when attempting to grab it from below. After the foreign object is removed, another checkup is needed to make sure there is no active bleeding, additional foreign bodies, or full-thickness injury to the bowel mucosa. Recovery state relies on daily monitoring of the patient status, physical examination to determine any trauma to the rectum or surrounding tissue, and follow-up to identify the appearance of new signs and symptoms.

## 4. Conclusion

The fast evaluation of the patient with a rectal foreign body was provided by using CT imaging. All the information provided helped in treatment decision. The clinical condition of our patient and the size and shape of the foreign body have allowed it to be grasped with the surgeon's hand and then be removed easily.

## References

[1] Rodríguez-Hermosa J. I., Codina-Cazador A., Ruiz B., Sirvent J. M., Roig J., Farrés R. (2007). Management of foreign bodies in the rectum. *Colorectal Disease*.

[2] Clarke D. L., Buccimazza I., Anderson F. A., Thomson S. R. (2005). Colorectal foreign bodies. *Colorectal Disease*.

[3] Aras M. H., Miloglu O., Barutcugil C., Kantarci M., Ozcan E., Harorli A. (2010). Comparison of the sensitivity for detecting foreign bodies among conventional plain radiography, computed tomography and ultrasonography. *Dento Maxillo Facial Radiology*.

[4] Yildiz S. Y., Kendirci M., Akbulut S., Ciftci A., Turgut H. T., Hengirmen S. (2013). Colorectal emergencies associated with penetrating or retained foreign bodies. *World Journal of Emergency Surgery*.

[5] Kokemohr P., Haeder L., Frömling F. J., Landwehr P., Jähne J. (2017). Surgical management of rectal foreign bodies: a 10-year single-center experience. *Innovative Surgical Sciences*.

[6] Cawich S. O., Thomas D. A., Mohammed F., Bobb N. J., Williams D., Naraynsingh V. (2016). A management algorithm for retained rectal foreign bodies. *American Journal of Men's Health*.

